# The Zinc–Senescence Axis in Skin Aging and Wound Healing: A Narrative Review

**DOI:** 10.3390/ijms27177713

**Published:** 2026-08-28

**Authors:** Kerim Mutig, Prim B. Singh, Dulat Azhibek, Svetlana Lebedeva

**Affiliations:** 1 Department of Biosciences, School of Medicine, Nazarbayev University, Astana 010000, Kazakhstan; kmutig@googlemail.com (K.M.); prim.singh@nu.edu.kz (P.B.S.); dulat.azhibek@nu.edu.kz (D.A.); 2 Scientific Center of Genetics and Life Sciences, Sirius University of Science and Technology, 119048 Sirius, Russia; 3 Department of Pharmacology, Institute of Pharmacy Named After A.P. Nelyubin, Sechenov First Moscow State Medical University, 119991 Moscow, Russia

**Keywords:** cellular senescence, SASP, skin aging, wound healing, inflammation, zinc

## Abstract

Approximately one billion people worldwide are affected by acute or chronic skin wounds, placing a substantial burden on healthcare systems. This burden is further increased by population aging, as wound healing is impaired in older individuals due to age-related changes in tissue repair. Structural and functional skin alterations associated with aging and wound healing have been extensively described, including cellular senescence and inflammaging. However, less is known about zinc homeostasis in aged skin and its impact on skin regeneration in aging. Zinc deficiency, which is common in older adults, may impair wound healing and contribute to the development of chronic wounds. The present review focuses on the age-related alterations of zinc homeostasis in the skin and the role of zinc in skin regeneration and wound healing.

## 1. Introduction

Wound healing remains a major global public health challenge and a considerable economic burden. In addition to surgical and traumatic wounds, the prevalence of other wound types, including pressure ulcers, venous ulcers, and diabetic foot ulcers, is increasing, largely as a consequence of population aging and the rising incidence of obesity and type 2 diabetes [1]. Wound healing is a highly coordinated process that proceeds through successive phases of hemostasis, inflammation, proliferation, and remodeling [2,3]. Zinc, as an essential trace element, is involved in each of these stages through its enzymatic, structural, and regulatory functions [4,5].

Zinc contributes to tissue repair, regeneration, differentiation, and re-epithelialization in multiple human tissues. Approximately one in ten human proteins binds zinc as a cofactor or structural component [5]. It is required for transcriptional regulation, deoxyribonucleic acid (DNA) repair, extracellular matrix (ECM) homeostasis, regulation of apoptosis, antioxidant defense, and metabolic regulation [6,7,8,9].

In plasma, Zn^2+^ concentrations range from 12 to 16 μM, although binding to plasma proteins such as albumin and α2-macroglobulin reduces the free, labile fraction to the nanomolar range [10]. Inside cells, metabolically active labile zinc is maintained at pico- to nanomolar concentrations, whereas extracellular levels are generally in the low nanomolar range [11]. Most of the body’s zinc is stored in skeletal muscle (60%), with additional substantial pools in bone (~30%) and in the skin and liver (5% each) [4].

At physiological concentrations, extracellular zinc binds to the G protein-coupled orphan receptor GPR39, which is widely expressed in the brain [12], colonocytes [13], keratinocytes [14], fibroblasts [15], osteoblasts [16], and cells of the pancreas, liver, gastrointestinal tract [17], prostate, and salivary glands [18]. The affinity of GPR39 for zinc is modulated by Ca^2+^, and the receptor itself mediates the link between changes in extracellular Zn^2+^ concentration and intracellular Ca^2+^ signaling.

Because zinc ions are hydrophilic, their movement across cell membranes is mediated by specific transport systems. The SLC39 (ZIP) family of importers increases cytosolic zinc availability by transporting zinc into the cytosol or releasing it from intracellular compartments, whereas the SLC30 (ZnT) family of exporters reduces cytosolic zinc by transporting it out of the cell or into organelles and zinc-storage compartments [19,20,21,22]. In addition to their subcellular distribution, ZIP and ZnT transporters exhibit tissue-specific expression profiles that correspond to the varying zinc demands across different organs [23]. Beyond these transporters, zinc homeostasis is regulated by metallothioneins (MT), which provide an intracellular zinc reservoir and control zinc release according to physiological demand. Zinc is also an integral component of matrix metalloproteinases (MMPs), integrins, and zinc-finger transcription factors, all of which participate in wound healing and dermal regeneration [24,25].

Zinc deficiency is associated with growth retardation, delayed puberty, impaired immune responses, inflammatory disorders, skin disease, and delayed wound healing [26]. Zinc deficiency is common worldwide and may arise from nutritional factors or genetic disorders such as acrodermatitis enteropathica. Older adults are a particularly vulnerable group. Although delayed wound healing is a recognized consequence of zinc deficiency, the role of zinc in wound repair, especially in the context of aging, remains incompletely understood. Therefore, a better understanding of the molecular, cellular, and physiological aspects of skin aging is essential for wound-care research and clinical practice. In this review, we discuss the contribution of zinc to wound healing, with particular emphasis on aging.

## 2. Zinc in Skin Physiology

The skin is the largest organ in the human body and the third most zinc-rich organ after muscle and bone. Overall, it contains approximately 5% of total body zinc; the epidermis contains about 50–70 µg/g, whereas the dermis contains about 10–50 µg/g [27,28]. Through its neuroendocrine system, the skin plays a key role in sensing environmental signals and orchestrating appropriate responses to maintain organismal homeostasis [29]. In addition, the skin fulfills protective and thermoregulatory functions, prevents transepidermal water loss, and contributes to vitamin D synthesis. It consists of three layers: the epidermis, dermis, and subcutaneous tissue (hypodermis) and contains approximately 20 different cell types with varying proliferative capacities.

### 2.1. The Epidermis

The epidermis is an avascular outer layer of the skin that serves as a barrier against contaminants, pathogens, dehydration, and ultraviolet (UV) radiation [30]. It is composed of keratinized stratified squamous epithelium comprising five major layers: stratum basale, stratum spinosum, stratum granulosum, stratum lucidum, and stratum corneum [31]. The epidermis consists primarily of keratinocytes at different stages of differentiation (90–95%), as well as melanocytes (3%), Langerhans cells (2%), T lymphocytes (0.3–1.3%) with immune functions, and Merkel cells (0.5%), which act as mechanoreceptors [30,31,32,33]. 

The outermost layer of the epidermis, the stratum corneum (horny layer), consists of dead, flattened corneocytes that maintain optimal skin hydration and serve as the primary barrier against external insults. Deep to the stratum corneum lies the stratum lucidum, a translucent layer composed of flattened corneocytes, which is present only in thick skin, followed by the stratum granulosum (granular layer), which contains keratinocytes undergoing terminal differentiation into corneocytes. The stratum spinosum contains mature keratinocytes with visible granules that are interconnected by desmosomes and actively produce keratin. The deepest layer of the epidermis, the stratum basale (or stratum germinativum), contains proliferating keratinocytes, melanocytes, Merkel cells, and epidermal stem cells.

At the base of the epidermis lies the basement membrane, where epidermal stem cells reside, ensuring the high proliferative capacity of the epidermis. Directly beneath the epidermis is the dermal-epidermal junction, which facilitates nutrient exchange, cellular signaling, and the removal of metabolic waste products from the epidermis [34]. The dermal-epidermal junction is characterized by an undulating contour formed by rete ridges, which are epithelial extensions that project the epidermis into the dermis and strengthen the connection between these two layers. The dimensions of rete ridges vary according to body site and age and increase during inflammatory skin diseases [35].

The epidermis generally contains a higher concentration of zinc than the dermis, with zinc being particularly enriched in the stratum spinosum. Keratinocyte proliferation occurs predominantly in the basal layer, whereas keratinocytes in the suprabasal layers progressively undergo differentiation. The dermis is a collagen-rich connective tissue in which fibroblasts are the principal resident cells. Zinc is required for normal keratinocyte proliferation and differentiation and may contribute to the regulation of cutaneous inflammation [36]. In particular, zinc has been reported to attenuate NF-κB activation and reduce the expression of pro-inflammatory mediators, including TNF and IL-1β [8]. In addition, zinc can suppress cytokine-induced inducible nitric oxide synthase (iNOS) expression and nitric oxide (NO) production, thereby potentially contributing to the limitation of inflammatory responses in the skin [37].

Epidermal development requires zinc-binding proteins, including enzymes and transcription factors. A key role is played by the p63 protein, a zinc-binding transcription factor that marks epidermal progenitor cells and regulates the cellular programs involved in epidermal formation [38]. Among zinc transporters expressed in the epidermis are ZIP1, ZIP6, and ZIP10. However, ZIP10 appears to be a key regulator of zinc homeostasis during epidermal development [39]. ZIP10 is highly expressed in hair follicles and basal epidermal cells. Experimental data suggest that ZIP10 may serve as the predominant zinc transporter responsible for zinc influx into epidermal precursor cells and may therefore participate in the development of the skin epithelium and its appendages [39]. During epidermal development, ZIP10 supplies zinc to p63, a master regulator of epidermal progenitor cell proliferation and differentiation, thereby enhancing its activity [39].

### 2.2. The Dermis

The dermis is the underlying layer of the skin that supports the epidermis and links it with the hypodermis. It performs several essential functions in maintaining skin structure and homeostasis. It provides mechanical support and elasticity through its rich ECM, which is composed primarily of collagen and elastic fibers, thereby conferring strength and resilience to the skin. In addition, the dermis fulfills a trophic function by supplying the avascular epidermis through its vascular and lymphatic networks, thus supporting nutrient delivery and metabolic exchange. It also plays a crucial role in thermoregulation through vascular responses and sweat gland activity and in sensory perception by housing nerve endings and specialized receptors responsible for detecting touch, pressure, pain, and temperature. Furthermore, the dermis contributes to cutaneous immune defense and wound repair, as it contains fibroblasts, immune cells, blood vessels, and signaling molecules that coordinate tissue regeneration and ECM remodeling.

The dermis is composed of two distinct layers: the upper papillary layer and the lower reticular layer [40]. The papillary layer is located superficially and is composed of loose fibrous connective tissue, whereas the reticular layer lies deeper and is formed by denser irregular connective tissue. The two layers differ in the organization of collagen fibers: in the papillary layer, type I collagen fibers are loosely arranged, whereas in the reticular layer they form large bundles [40]. 

Collagen is one of the most essential proteins in the skin, accounting for approximately 75% of its dry mass. To date, approximately 28 genetic types of collagen have been identified [41], and their expression changes throughout life [42]. In addition, the dermis contains distinct types of elastic fibers, including elastin, fibrillin microfibrils, elaunin fibers, and oxytalan fibers [43]. The dermis also contains hair follicles, sweat glands, sebaceous glands, lymphatic vessels, nerves, and blood vessels. It is enriched with immune cells, including macrophages, dendritic cells, natural killer (NK) cells, T and B cells, and mast cells [44]. 

The main cells of the dermis are fibroblasts, which synthesize collagen, elastin, and the ground substance of the ECM. The ECM is composed of type I collagen, elastic fibers, proteoglycans, and hyaluronic acid (HA). The dermal ECM plays vital roles in structural support, immunity, circulation, and sensory perception.

The key biological properties of the dermis are determined by adhesion proteins (fibronectin, laminin, and fibrillin), glycosaminoglycans (HA, heparan sulfate, and chondroitin sulfate), and proteoglycans.

In the dermis, zinc is present at higher concentrations in the upper layers than in the lower layers, owing to the greater abundance of zinc-rich mast cells, which are required for cytokine production [45]. Dysregulation of zinc levels in the skin impairs fibroblast function, leading to ECM defects and delayed tissue repair. Zinc homeostasis in fibroblasts is regulated by ZIP4, ZIP6, ZIP7, ZIP10, ZIP13, and ZIP14 [46].

### 2.3. The Hypodermis

The hypodermis, or subcutaneous tissue, is the deepest layer of the skin, anchoring the dermis to the underlying muscles. This tissue consists of adipocytes, large blood vessels, nerves, and adipose stromal cells, as well as nearly all types of immune cells, including mast cells, macrophages, memory T cells, and Langerhans cells [47]. The hypodermis plays a key role in thermoregulation, protects blood vessels, nerves, muscles, and bones, serves as a reservoir of progenitor cells, and participates in paracrine and endocrine signaling, as well as energy storage [48,49]. Mesenchymal stem cells of the hypodermis play a crucial role in wound healing and re-epithelialization [50].

## 3. Zinc and Mechanisms of Skin Aging

The skin provides a physical barrier against the external environment and is therefore permanently exposed to environmental aggressors and internal stressors. Together with chronological aging, these factors drive skin aging, a multifactorial biological process characterized by phenotypic alterations and a decline in physiological function [29]. Skin aging leads to dysregulation of cellular processes and loss or fragmentation of ECM fibers, which is clinically manifested as wrinkles, laxity, and pigmentary changes.

### 3.1. Molecular Mechanisms of Skin Aging

**Figure 1:**
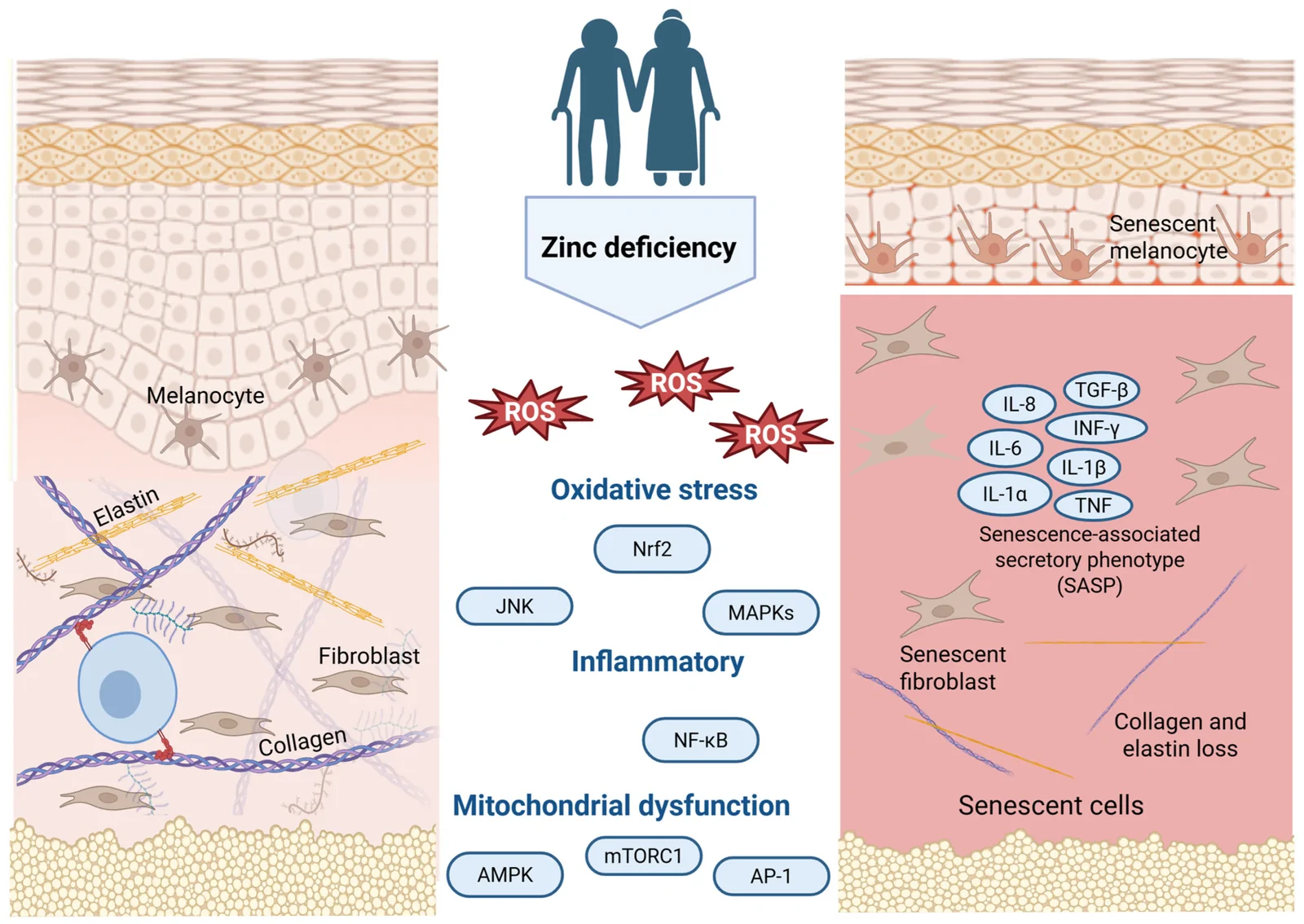
Age-related skin changes. Aging is associated with a higher risk of zinc deficiency. The skin is exposed to excessive reactive oxygen species (ROS), leading to oxidative stress, inflammation, and mitochondrial dysfunction. Senescent cells acquire a senescence-associated secretory phenotype (SASP) characterized by the release of pro-inflammatory cytokines. The epidermis becomes thinner, the stratum spinosum atrophies, and the number of melanocytes decreases. The dermoepidermal junction flattens, collagen and elastin fibers are lost, and the volume of subcutaneous white adipose tissue (sWAT) decreases.

With age, the skin undergoes metabolic alterations and a progressive decline in endogenous antioxidant defenses, which increases its susceptibility to external stressors, particularly UV radiation, environmental pollution, and microbial exposure [51]. Excessive production of reactive oxygen species (ROS), resulting from age-related loss of antioxidant capacity, activates mitogen-activated protein kinase (MAPK) pathways, the PI3K/mTOR/S6K signaling axis, and transcription factors such as nuclear factor-κB (NF-κB), nuclear factor erythroid 2-related factor (Nrf2), c-Jun N-terminal kinase (JNK), and activator protein 1 (AP-1), thereby amplifying oxidative stress and inflammatory signaling [52,53,54]. At the molecular level, skin aging is associated with reduced expression of mitochondrial electron transport chain proteins, accumulation of damaged mitochondria, and fragmentation of the mitochondrial network. Mitochondrial dysfunction is integrated through signaling pathways involving AMP-activated protein kinase (AMPK), mammalian target of rapamycin (mTOR), and calcium-dependent regulators [55]. Notably, mammalian target of rapamycin complex 1 (mTORC1) serves as a central signaling hub that integrates diverse stress cues and regulates cell growth, and its activation has been linked to stimuli that promote cellular senescence [56] (Figure 1). 

In addition to increased ROS production and mitochondrial dysfunction, senescent cells display profound changes in chromatin organization and gene expression and acquire a distinctive secretory phenotype known as the senescence-associated secretory phenotype (SASP). SASP comprises a broad spectrum of pro-inflammatory cytokines, chemokines, growth factors, and other bioactive mediators, including interleukin-6 (IL-6), IL-1α, IL-1β, IL-8, tumor necrosis factor (TNF), interferon-γ (IFN-γ), and transforming growth factor-β (TGF-β), which together contribute to chronic low-grade inflammation, tissue dysfunction, and age-related pathology [57,58,59,60]. In addition, SASP enables senescent cells to activate, suppress, modulate, and/or evade the immune system [60]. 

It has been shown that SASP production is amplified by NF-κB signaling [61] and contributes to the establishment of a pro-inflammatory microenvironment in tissues [62] and to the development of chronic low-grade inflammation [57,63], while also representing one of the major drivers of tissue homeostatic dysregulation [59]. The presence of inflammatory markers in aging skin may indicate a more complex dysfunction of the immune system, even when it is restricted to local manifestations. 

Through paracrine signaling, SASP also alters the microenvironment of neighboring cells, suppresses ECM production, and promotes the spread of senescence within tissues [64,65]. This phenomenon has been elegantly demonstrated in heterochronic parabiosis experiments, which showed that circulating factors from young and old systemic environments exert pro-youthful and pro-aging effects, respectively, although the precise nature of these circulating mediators remains incompletely understood [66,67]. 

Since the discovery of cellular senescence by Hayflick and Moorhead in 1961 [68], the scientific community has sought universal markers capable of reliably defining the senescent state. However, the identification of such markers remains challenging due to the complexity of the senescence phenotype and the existence of highly heterogeneous senescence programs [69]. Accordingly, the use of combined marker panels, together with SASP factor expression, has been recommended for the accurate identification of senescent cells [70]. Among the most widely used markers are p16I^NK4a^, p14ARF/p19ARF, and p21^CIP1/WAF1^, which function as cell cycle inhibitors [71,72]. Of these, p16^INK4a^ is considered one of the most informative and intensively studied markers of cellular senescence [73]. Additional biomarkers include increased SA-β-galactosidase activity [74], altered lamin B1 levels [75], and telomere-associated foci [76].

### 3.2. Cellular Mechanisms of Skin Aging

Cellular senescence is a protective response to molecular damage that accumulates with aging [77]. Cells can undergo senescence in response to a variety of stressors, which are commonly grouped into four major categories: (1) DNA damage; (2) metabolic stress, including high glucose, osmotic stress, oxidized or saturated fatty acids, ceramides, and ROS; (3) inflammation; and (4) damage signals, such as damage-associated molecular patterns (DAMPs), including nucleotides, urate, chromatin fragments, misfolded or aggregated proteins, and related factors [60,78]. 

Cellular senescence occurs in all layers of the skin. Between the ages of 30 and 70 years, epidermal cell turnover declines markedly [79,80]. This leads to a reduction in the number of melanocytes and Langerhans cells, which process microbial antigens and present them to other immune cells. These changes are accompanied by alterations in T- and B-cell function and by a pro-inflammatory state known as inflammaging [81]. The dermal-epidermal junction becomes flatter, and the papillary dermis is smoothed, resulting in reduced contact between the dermis and epidermis. This impairs nutrient exchange and the removal of metabolic waste products, thereby contributing to their accumulation and increased ROS production [80]. As epidermal turnover declines, desquamation becomes less efficient and wound healing is impaired [58].

***Keratinocytes.*** Senescent keratinocytes undergo molecular and functional changes associated with features of intrinsic aging and exhibit signs of increased autophagic activity [82]. During skin aging, keratinocyte differentiation is impaired, their number decreases, and the expression of adhesion molecules and intercellular junction proteins is reduced. These changes lead to epidermal thinning, reduced barrier function, diminished protection against environmental insults, and a decreased ability to restore the skin barrier after injury [59]. Given the important role of keratinocytes in skin barrier formation and cytokine production, and therefore in inflammatory processes, dysregulation of these cellular pathways may be a key contributor to skin aging [59]. At the same time, the notion that keratinocytes can acquire senescent phenotypes has been questioned, given their highly proliferative nature [83].

***Melanocytes.*** Melanocytes are the main senescent cell population in the aging epidermis [76]. For most of their lifespan, they remain in a quiescent state and can re-enter the cell cycle in response to signals from neighboring keratinocytes [84]. Studies have shown that p16^INK4a^ expression is increased in aging melanocytes [58,85] and that the senescent phenotype of these cells is acquired mainly through telomere damage that is independent of telomere length [29]. Moreover, melanocyte telomere damage foci have been positively correlated with age-related skin features, such as flattening of the dermal-epidermal junction [29]. Molecular alterations in melanocytes of aging skin lead to visible hypo- or hyperpigmented areas, as well as other pigmentary disorders associated with impaired proteasomal activity and enhanced autophagy [86]. Melanocyte aging is also characterized by melanin accumulation due to defective melanosome transport and activation of glycolytic pathways [87]. Fibroblasts accumulating in pigmented areas may further stimulate melanogenesis. Overall, the contribution of senescent melanocytes to age-related skin changes appears to be primarily related to pigmentation. 

***Fibroblasts.*** With age, both the number and functional capacity of fibroblasts decline synchronously [88]. Fibroblasts are the principal cellular component of connective tissue and are essential for the synthesis, organization, and remodeling of collagen; they also play a critical role in maintaining ECM integrity. They are particularly important in wound healing, where they proliferate at the injury site and deposit collagen to form a provisional matrix [89]. In aging fibroblasts, protein synthesis, folding, and degradation, as well as post-translational modifications, become dysregulated, leading to proteostasis impairment and the accumulation of senescence biomarkers such as senescence-associated β-galactosidase (SA-β-gal), p16I^NK4a^, p21^CIP1/WAF1^, and p53 [58,77,90]. Similar to melanocytes, senescent fibroblasts show increased glycolysis and reduced lamin B1 expression [59]. In addition, senescent fibroblasts express MMP-1 [91] and MMP-9 [92]—key mediators of skin aging that promote collagen degradation, epithelial–mesenchymal transition, angiogenesis, and inflammation [93].

Fibroblasts in aged skin exhibit a collapsed cytoplasm and a rounded morphology, in contrast to the spread morphology of fibroblasts in young skin [94]. These cells show a reduced capacity to synthesize type I collagen, resulting in a shift in collagen production from type I to type III [95]. Aging skin is also characterized by fragmentation of dermal collagen and elastin, which contributes to reduced elasticity and turgor [96].

In senescent fibroblasts, TGF-β–mediated signaling is attenuated, further decreasing collagen synthesis. Collagen becomes disorganized and less densely packed. In addition, aging fibroblasts display reduced migratory capacity along the surrounding collagen network [97], while increased MMP expression impairs their adhesion to the ECM and reduces cellular extensibility.

The accumulation of senescent dermal fibroblasts, which lose their cellular identity, decrease collagen production, and exhibit enhanced SASP release, markedly accelerates skin aging [98]. 

Collectively, the cumulative effects of aging, stress, and SASP signaling in fibroblasts contribute to age-related skin changes, including wrinkle formation, loss of ECM components such as collagen and elastin, systemic inflammation, and disease-associated alterations [98]. 

***Adipose tissue.*** Adipose tissue of the hypodermis undergoes significant structural changes with age, including redistribution of fat depots: the overall volume of subcutaneous white adipose tissue (sWAT) decreases, whereas visceral adipose tissue volume increases. Progressive loss of subcutaneous fat leads to reduced mechanical support of the skin, decreased skin tension, the development of sagging, and wrinkle formation [99]. Age-associated dysfunction of the hypodermis contributes to the acceleration of skin aging processes, including chronic low-grade inflammation, immunosenescence, delayed wound healing, and impaired intercellular interactions.

Thus, skin aging represents a complex of cumulative biochemical, mechanical, and environmental changes that accumulate over time. These processes result in both pronounced morphological alterations and functional impairments associated with reduced regenerative and reparative capacity of the skin. Characteristic phenotypic features include epidermal thinning, decreased collagen content, and disorganization of elastic fibers, which collectively lead to atrophy, increased fragility, and wrinkle formation. Overall, age-related changes disrupt the structural organization and biomechanical properties of the skin, increasing its susceptibility to mechanical damage and slowing wound healing processes [100]. As a result, elderly individuals have an elevated risk of injury, wound infection, and the development of chronic wounds [100,101].

## 4. Zinc in Skin Wound Healing

### 4.1. Phases of Wound Healing

Cutaneous wound healing is a complex biochemical signaling cascade comprising four main phases: hemostasis (from injury to several hours thereafter), inflammation (1–3 days), proliferation (4–21 days), and ECM remodeling (from 21 days up to 1 year) [102,103]. 

Following vascular injury, the hemostatic phase begins immediately: vasoconstriction of the damaged vessels and platelet aggregation lead to the formation of a platelet plug, while activation of the coagulation cascade results in a fibrin clot that stops bleeding and provides a provisional matrix for subsequent cell migration. Activated platelets and damaged endothelial cells release cytokines and growth factors that initiate the recruitment of inflammatory cells [104]. Concurrently, vascular endothelial growth factor (VEGF) increases vascular permeability, leading to the formation of primary edema. This is subsequently followed by secondary edema, driven by increased oncotic and osmotic pressure within tissues, as well as fluid redistribution into the interstitial space [105]. These processes initiate the inflammatory phase, which aims to prevent physiological disturbances, initially by restoring hemostasis and counteracting pathogens and subsequently by promoting tissue repair. During the inflammatory phase, neutrophils are the first leukocytes to infiltrate the wound [106], followed by macrophages, which clear cellular debris and bacteria, release further growth factors, and orchestrate the transition to tissue repair [107].

The proliferative phase comprises three key processes: re-epithelialization, which involves keratinocyte proliferation and migration across the wound bed; granulation tissue formation, characterized by fibroblast proliferation, migration, and ECM synthesis; and neovascularization. During granulation, fibroblasts migrate into the fibrin clot, where they proliferate and synthesize collagen type I and III, fibronectin, and proteoglycans [108], thereby facilitating keratinocyte migration into the granulation tissue. The remodeling phase involves the replacement of granulation tissue with structurally and functionally mature skin. This process is considerably longer than the preceding stages and may last up to 6 months. The final stage of wound healing restores the barrier function of the skin through complete re-epithelialization.

**Figure 2:**
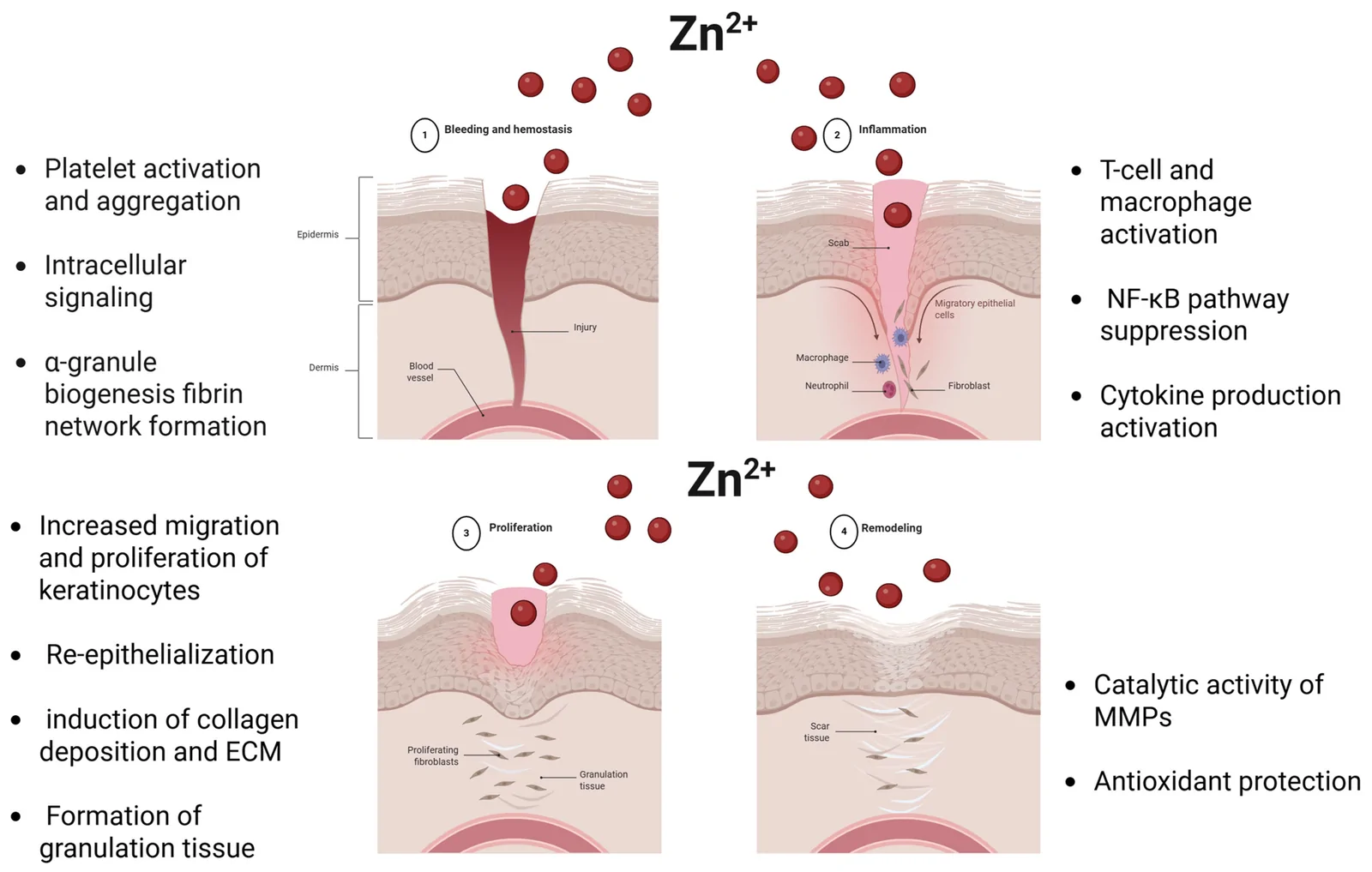
The role of zinc across the different stages of wound healing.

The duration of each phase depends on multiple factors, including wound type and size, patient age, overall health status, comorbidities, wound location, and treatment strategy. All stages of wound healing are mediated by the coordinated activity of various cell types (platelets, leukocytes, keratinocytes, fibroblasts, epithelial cells, and stem cells) and biologically active molecules (cytokines, growth factors, ROS, and ECM proteins) operating in a highly intricate sequence [109]. Notably, the essential trace element zinc plays a critical role at all stages of wound healing (Figure 2).

Nearly half a century ago, it was reported that zinc concentrations increase by approximately 20% in regenerating tissue compared with intact skin [110]. Zinc deficiency may exacerbate inflammatory skin disorders and lead to delayed wound healing. In this context, elevated levels of free zinc ions and increased ZnT1 expression in senescent fibroblasts [111] suggest that zinc dyshomeostasis contributes to the development of a pro-inflammatory secretory phenotype. This association between zinc metabolism and cellular senescence highlights the potential role of altered zinc homeostasis in promoting inflammaging [62]. 

### 4.2. Role of Zinc at Different Stages of Wound Healing

***Hemostasis.*** The role of zinc in platelet activation and aggregation has long been underestimated. However, as early as the mid-20th century, zinc was identified as an important hemostatic factor that enhances platelet activation and aggregation and acts synergistically with calcium [112]. 

Elevated zinc concentrations in atherosclerotic plaques [113] and at sites of vascular injury—resulting from its release from platelet granules—underscore its function as a transmembrane signaling mediator during platelet activation and thrombus formation [114]. Zinc deficiency leads to prolonged bleeding time in both animal models [115,116,117] and humans [118].

At a concentration of 100 μM, Zn^2+^ acts as a platelet agonist by modulating intracellular signaling pathways, leading to granule secretion, protein kinase C (PKC)-dependent tyrosine phosphorylation, activation of integrin αIIbβ3, and platelet aggregation. At lower concentrations (30 µM), zinc functions as a co-activator, enhancing platelet aggregation. Due to active transport mechanisms, platelets can take up Zn^2+^ from plasma and accumulate it in significant amounts within α-granules. To maintain zinc homeostasis, various ZIP and ZnT transporters are expressed in megakaryocyte membranes [119,120]; however, further studies are needed to elucidate the molecular mechanisms underlying zinc uptake, storage, and release in platelets, as well as the specific contributions of individual ZIP/ZnT isoforms to zinc homeostasis [120]. 

Taylor and Pugh (2016) proposed a model describing zinc-dependent mechanisms of platelet activation [118]. According to this model, vascular injury and initial collagen-induced platelet activation lead to a marked increase in intracellular Zn^2+^ levels. This increase is thought to result from the activity of membrane ion channels and transporters, as well as partial release from intracellular stores. Supporting this model, recent studies have shown that Zn^2+^ influx into platelets and subsequent activation are associated with transient receptor potential (TRP) channels, the Na^+^/Ca^2+^ exchanger (NCX1), and ZIP7 [121].

In addition to α-granules, zinc is present in the platelet cytosol in both metallothionein-bound and free forms. Following vascular injury, Zn^2+^ released from platelets increases the affinity of the glycoprotein VI (GPVI) receptor for collagen, thereby inducing primary platelet activation, thromboxane A_2_ (TxA_2_) release, and α-granule secretion [112]. Interaction of zinc with PKC promotes tyrosine phosphorylation of signaling proteins and subsequent conformational changes in glycoprotein IIb/IIIa (GPIIb/IIIa), facilitating fibrin binding and thrombus formation.

Zinc ions regulate several steps of the coagulation cascade: they bind to factor XII, inducing conformational changes, and interact with fibrinogen, high-molecular-weight kininogen (HMWK), and histidine-rich glycoprotein (HRG). Zinc-induced conformational changes in factor XII activate the kallikrein–kinin system, leading to bradykinin release and accumulation on the endothelial surface, which promotes the adhesion of inflammatory immune cells. Zinc deficiency has been shown to impair the coagulation cascade and fibrin formation, resulting in prolonged bleeding time [118]. 

Zinc also regulates the biogenesis and release of multiple α-granule components, including fibrinogen, prothrombin, coagulation factors V, XI, and XIII, von Willebrand factor (VWF), fibronectin, P-selectin (CD62P), platelet-derived growth factor (PDGF), epidermal growth factor (EGF), β-thromboglobulin, albumin, kallikrein, and α_2_-antiplasmin [118].

Thus, zinc contributes to hemostasis through multiple mechanisms that regulate platelet activation, coagulation, and fibrin network formation [120]. Nevertheless, further studies are required to clarify the pathways of Zn^2+^ entry into the platelet cytosol and the downstream zinc-dependent signaling mechanisms.

***Inflammation.*** The inflammatory phase of wound healing is directed toward the restoration of damaged tissue through the activation of cells and the release of inflammatory mediators, whose interactions drive both local and systemic acute inflammatory responses. During this phase, keratinocytes rapidly migrate into the wound bed and initiate re-epithelialization, which is stimulated by modified ECM proteins and cytokines [122]. The principal producers of cytokines are macrophages and other immune cells [123].

Zinc is an essential component of thymulin, a thymopoietic hormone required for the proliferation and differentiation of T helper cells [124]. Zinc influx from extracellular and intracellular sources via ZIP6 and ZIP8 transporters, respectively, contributes to T-cell activation [125]. ZIP6-mediated increases in intracellular zinc promote the differentiation of CD4 T cells into several subsets, including Th1, Th2, Th17, and T regulatory (Treg) cells [125]. An increase in zinc levels mediated by ZIP8 inhibits calcineurin activity, thereby promoting cAMP response element-binding protein (CREB) phosphorylation and IFN-γ transcription [125]. In addition, ZIP8 is a transcriptional target of NF-κB and acts as a negative regulator of pro-inflammatory responses by zinc-dependent downregulation of IκB kinase (IKK) activity in vitro [126]. Increased zinc levels associated with ZIP8 upregulation in monocytes, macrophages, and dendritic cells negatively regulate NF-κB signaling [127], whereas zinc deficiency, conversely, promotes excessive inflammation [126]. ZIP10 is essential for B-cell survival and proper function in humoral immunity [128].

Zinc likely influences cytokine production by increasing the expression of peroxisome proliferator-activated receptors (PPARs) [129,130] and zinc-finger protein TNF-α-induced protein 3 (TNFAIP3, also known as A20), both of which suppress NF-κB signaling [127].

It is now well established that zinc participates in macrophage activation and is required for both degranulation and cytokine production [15,131,132]. Moreover, macrophages may be among the key cells through which zinc regulates wound healing, potentially by promoting polarization from the M1 phenotype to the M2 phenotype [133,134,135]. Zinc deficiency adversely affects the expression of IL-2 and IFN-γ and increases the production of the pro-inflammatory cytokines IL-1β, IL-6, and TNF [136], which are involved in wound healing, likely through GPR39-mediated signaling pathways [15].

***Proliferation.*** For wound healing, it is important to suppress the inflammatory response as early as possible and prevent its progression to a chronic state. Treg cells contribute to the resolution of inflammation as well as to re-epithelialization and wound contraction [123,137]. Several studies have shown that zinc supplementation, by modulating Treg signaling pathways, promotes their induction and stability [138,139,140]. Under the action of collagenases, plasminogen activators, and zinc-dependent MMPs, the fibrin clot is degraded, thereby creating space for cell proliferation, migration, and angiogenesis. The X-linked zinc finger protein (ZFX) is also known to promote keratinocyte proliferation and migration [141]. In addition, zinc is an essential cofactor for the proper function of SMAD proteins, which serve as key mediators of signaling from TGF-β receptors. The increase in zinc concentration from 8 h to 3 days after wounding, as well as the high expression of GPR39 and ZIP4 in keratinocytes, indicates an important role of zinc in proliferative processes [142,143]. 

During keratinocyte proliferation, the content of MTs, which are redox-sensitive antioxidant proteins, increases, and this is important for wound healing. It has been shown that approximately 20% of intracellular zinc is bound to MTs [4], which regulate its distribution in accordance with physiological requirements [144]. At the same time as re-epithelialization, endothelial cells migrate to and proliferate at the wound site, forming new blood vessels. In this way, newly formed cells are supplied with oxygen and nutrients necessary for their survival and growth [145]. It is known that expression of the zinc-dependent protein ZEB2 is increased in injured cardiomyocytes. Delivery of a therapeutic dose of ZEB2 into cardiomyocytes induces angiogenesis and increases the density of the newly formed vascular network, resulting in reduced scar formation and preservation of cardiac function [146]. However, the exact role of zinc in regulating angiogenesis remains poorly understood.

***Remodeling.*** The remodeling phase begins after completion of re-epithelialization and is directed toward replacing granulation tissue with more mature and functionally competent skin [143]. This stage represents a dynamic equilibrium between tissue synthesis and degradation, regulated by proteolytic enzymes. A key process in remodeling and tissue restoration is the degradation of various proteins of the ECM mediated by MMPs. MMPs belong to the family of zinc-dependent endopeptidases identified in diverse tissues [147] and are secreted by various cell types, including inflammatory cells, keratinocytes, endothelial cells, fibroblasts, vascular smooth muscle cells (VSMCs), and others [148]. All members of the MMP family contain catalytic domains in which zinc is essential for proteolytic activity [149,150]. In the skin, MMPs participate in numerous cellular, molecular, and biochemical processes, modulating the release of cytokines, growth factors, and other bioactive agents sequestered in the ECM [151,152]. In human acute wound models, formation of neo-epithelium is impaired when MMP activity is inhibited [153]. Mouse and cellular model studies have demonstrated that MMP-1, MMP-7, and MMP-9 contribute to re-epithelialization and are activated during wound healing [154,155]. 

MMPs can hydrolyze virtually all ECM proteins, including collagen and elastin, thereby contributing to the structural organization and regeneration of the dermis and epidermis [156]. They also participate in vascular remodeling, cell growth, migration, and differentiation, as well as tissue invasion and vascularization [157]. Additionally, MMPs can affect endothelial cell function, VSMC migration, proliferation, Ca^2+^ signaling, and muscle contraction [149,152]. Triggers for MMP activation include tissue injury, oxidative stress, inflammatory cytokines, hormones, growth factors, and UV radiation [150]. Among the MMP family enzymes, collagenases are particularly important due to their ability to hydrolyze native collagen. These collagenolytic enzymes are effective proteolytic complexes because they degrade collagen, the main component of wounds and scars. Studies have shown that collagenases influence reparative processes, including cell migration, proliferative and angiogenic responses to injury in vitro, and enhanced wound healing in vivo [158]. Thus, during the remodeling phase, wound re-epithelialization is completed, and the dermis gradually restores its strength and mechanical resilience.

Overall, zinc plays an essential and multifaceted role in wound healing across all phases, although its precise mechanisms remain incompletely understood.

### 4.3. Zinc and GPR39

Following skin injury, extracellular Zn^2+^ is released and acts as a first messenger, inducing intracellular Ca^2+^ signaling via the Gαq-coupled Zn^2+^-sensing receptor mZnR/GPR39 [159]. The resulting Ca^2+^ increase activates ERK/MAPK and PI3K/AKT pathways in keratinocytes, promoting cell survival and proliferation. In addition to keratinocytes, GPR39 expression is upregulated in fibroblasts and the hypodermis, underscoring a direct role for Zn in wound repair via mZnR/GPR39 [15]. Activation of mZnR/GPR39 also stimulates the Na^+^/H^+^ exchanger (NHE1), leading to extracellular acidification. This extracellular acidification may facilitate keratinocyte migration and barrier formation [160]. The acidic environment of the skin’s superficial layer is essential for permeability barrier formation, whereas zinc deficiency exacerbates inflammatory responses [161]. For keratinocytes, NHE-mediated stabilization of cytosolic pH is critical, as it supports migration, proliferation, and responses to tissue injury. In the context of wound healing, GPR39 may act as an amplifier of the reparative response: it stimulates keratinocyte proliferation and concurrently helps cells manage metabolic acid load through NHE-dependent pH regulation.

Conversely, under conditions of acidosis or alkalosis, GPR39-dependent activation of NHE1 is attenuated, protecting cells from excessive decreases or increases in pH. Thus, mZnR/GPR39 signaling provides a homeostatic adaptive mechanism that regulates changes in both intracellular and extracellular pH [162]. Notably, ZnR/GPR39 exerts paracrine effects on adjacent cells, suggesting a mechanism by which Zn^2+^ regulates tissue responses even in cells lacking receptor expression [159].

In addition, GPR39 activation can lead to adenosine triphosphate (ATP) release [163] in keratinocytes and, via paracrine signaling, increase the proliferation of neighboring fibroblasts.

**Figure 3:**
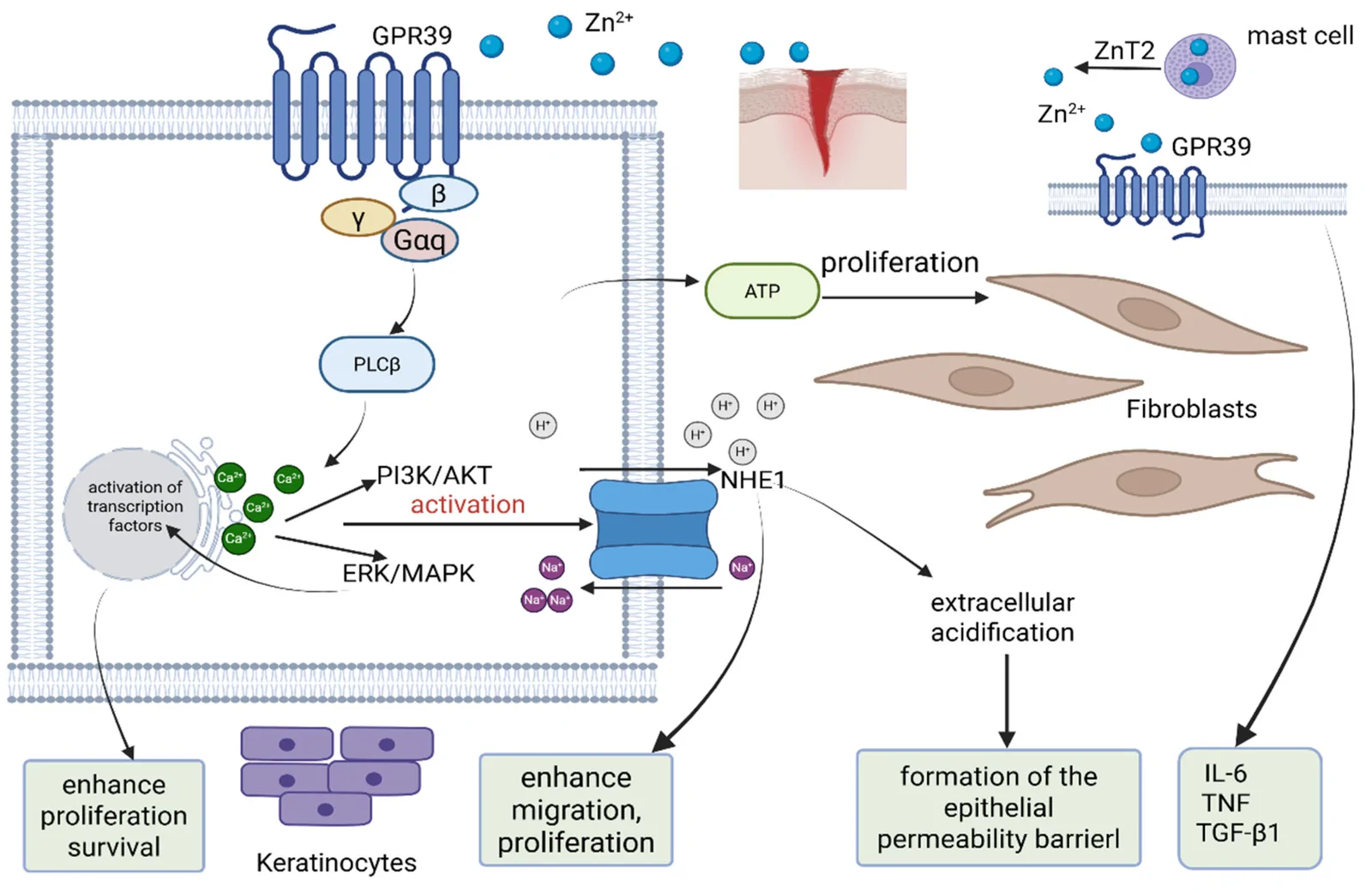
Zinc-dependent regulation of GPR39 in wound healing. Following skin injury, Zn^2+^ accumulates in wound tissues and activates the Gαq-coupled Zn^2+^-sensing receptor GPR39 on the keratinocyte membrane. Activation of Gαq triggers PLCβ activation and subsequent Ca^2+^ release from ER stores. The resulting intracellular Ca^2+^ rise activates PKC and PI3K pathways, which may enhance keratinocyte proliferation. Activation of GPR39 by extracellular Zn^2+^ stimulates the Na^+^/H^+^ exchanger (NHE1) in keratinocytes via the MAPK signaling pathway, thereby accelerating proton efflux from the cell. Stabilization of cytosolic pH is essential for keratinocyte migration, proliferation, and responses to tissue injury. GPR39 activation can lead to ATP release in keratinocytes and, via paracrine signaling, increase the proliferation of neighboring fibroblasts. Zinc is released from mast cell granules via ZnT2 and increases the mRNA expression of IL-6, TGF-β1, TNF, and other inflammatory cytokines.

According to recent findings, mast cells may promote wound healing through ZnT2-dependent Zn^2+^ release and subsequent GPR39-mediated cytokine induction in dermal fibroblasts [15] (Figure 3). 

## 5. Age-Related Changes in Wound Healing

Cellular senescence plays an evolutionarily advantageous role by facilitating tissue remodeling during development and after injury. It also contributes to wound healing, where a transient induction of senescent cells occurs during normal acute repair and can be beneficial [164]. In a mouse model, the appearance of senescent fibroblasts and endothelial cells was shown to stimulate myofibroblast differentiation through secretion of platelet-derived growth factor AA (PDGF-AA), a key SASP-associated factor [164]. Furthermore, SASP derived from senescent cells can promote keratinocyte regeneration and the acquisition of stem-like characteristics. 

However, acute and chronic senescence can mediate opposite effects, as confirmed in various wound-healing models [165,166]. Cellular senescence may impair tissue regeneration by inducing inflammation and fibrosis [69]. Retention of senescent cells at later stages of wound healing leads either to chronic non-healing wounds, as seen in diabetes, or to excessive ECM deposition and fibrosis development [57]. In a mouse model with delayed diabetic wound healing, an increased number of senescent cells was observed, with macrophages representing a significant proportion [167]. In elderly individuals, wounds heal more slowly and are typically accompanied by complications [168], and regenerated tissues exhibit reduced strength compared to those of young individuals [169,170]. Combined with an impaired immune system, this contributes to increased risk of secondary infection in elderly patients. Elevated levels of inflammatory cytokines and altered MMP levels and tissue MMP inhibitors after wound healing can result in incomplete healing in elderly individuals and recurrence of chronic wounds.

Age-related differences are evident at each stage of wound healing. Increased levels of factors influencing platelet activation and blood coagulation, along with accumulation of collagen cross-links, generally lead to reduced bleeding time. The inflammatory phase of wound healing occurs later in aged mice, possibly due to decreased glycosaminoglycan content, variations in mechanical stiffness and mechanotransduction, and declining macrophage function [100]. Reduced levels of hyaluronic acid, which regulates cell proliferation and migration in the epidermis and dermis and maintains a hydrated matrix facilitating migration and re-epithelialization after injury, also delay the onset of the inflammatory phase [171,172].

Oxidative stress plays a key role in wound healing. Imbalance between free radicals and antioxidants in the body leads to excessive ROS production, causing cellular and tissue damage and delaying wound healing [173]. ROS play a pathological role at all stages of wound regeneration. Specifically, during the inflammatory phase, large amounts of ROS, along with pro-inflammatory cytokines and proteolytic enzymes, are released by neutrophils and macrophages. ROS also participate in epithelialization by activating EGF and keratinocyte growth factor (KGF) receptors and inducing TGFα production in fibroblasts. The role of ROS signals in angiogenesis is now well understood. Moderate H_2_O_2_ levels regulate VEGF production and accelerate angiogenesis. Conversely, excessive ROS levels inhibit angiogenesis. Overall, excessive ROS indicates unbalanced redox homeostasis and impairs wound healing.

The antioxidant action of zinc occurs through multiple mechanisms: competition with iron and copper ions for binding to cellular membranes and proteins, displacing these redox-active metals; binding to sulfhydryl (–SH) groups of molecules, protecting them from oxidation; activation of antioxidant proteins, molecules, and enzymes, including glutathione, catalase, and superoxide dismutase; binding to MTs; stabilization of cellular membranes; and regulation of antioxidant enzyme gene expression through zinc-dependent transcription factors. These mechanisms provide comprehensive cellular protection against oxidative stress, which is particularly important during wound healing, where oxidative stress can disrupt reparative processes.

## 6. Clinical Implications of Zinc Supplementation

The clinical implications of zinc supplementation are particularly relevant for older adults and patients with chronic or delayed-healing wounds. Individuals with documented zinc deficiency, inadequate dietary intake, malabsorption, chronic inflammation, diabetes, or increased metabolic requirements may represent the population most likely to benefit from zinc replacement. In these patients, restoration of zinc availability may support keratinocyte proliferation and migration, fibroblast activity, immune regulation, antioxidant defense, and ECM remodeling. Zinc supplementation may therefore have potential value as an adjunctive intervention in the management of pressure ulcers, diabetic foot ulcers, postoperative wounds, and other chronic cutaneous lesions. 

In a randomized, double-blind, placebo-controlled trial involving 60 patients aged 40–85 years with grade 3 diabetic foot ulcers, daily supplementation with 220 mg of zinc sulfate, providing 50 mg of elemental zinc, for 12 weeks was associated with a significant reduction in ulcer length and improvements in metabolic parameters [174]. A double-blind, randomized study of 30 patients showed that intravenous zinc supplementation (30 mg/day for 3 days) corrected the postoperative decline in serum zinc, particularly in its non-α2-macroglobulin-bound fraction, and was associated with improved wound healing [175]. 

However, the clinical benefit is unlikely to be uniform across all patients and may depend on the baseline zinc status, cause and severity of the wound, nutritional condition, comorbidities, and route of administration. Importantly, the molecular and cellular evidence supporting the role of zinc in tissue repair does not necessarily translate into a predictable therapeutic effect of systemic supplementation in individuals with adequate zinc status.

Both systemic and topical zinc formulations may be clinically relevant. Oral supplementation primarily aims to correct systemic deficiency, whereas topical preparations may provide a more direct effect at the wound site. Nevertheless, differences in zinc salts, concentrations, formulation properties, tissue penetration, and treatment duration make it difficult to compare available studies. Zinc supplementation should therefore be considered an adjunct to, rather than a replacement for, standard wound care, including debridement, infection control, pressure relief, vascular assessment, glycemic control, and adequate protein-energy nutrition.

Before routine clinical use, further well-designed clinical trials are needed to determine the optimal dose, formulation, duration of treatment, and target patient population. Future studies should incorporate baseline and post-treatment zinc status, standardized wound-healing outcomes, assessment of nutritional status, and clinically meaningful endpoints such as time to complete wound closure, infection rate, recurrence, and scar quality.

### 6.1. Limitations

This article is a narrative review and therefore does not follow a fully systematic literature-search strategy. The selection of studies was based on their relevance to the scope of the review and the authors’ expert judgment. Accordingly, the methodological quality and risk of bias of the included studies were not formally assessed. These features should be considered when interpreting the conclusions of this review.

### 6.2. Conclusions

Zinc is a key regulator of skin homeostasis and repair, playing essential roles in keratinocyte proliferation and differentiation, fibroblast function, immune regulation, antioxidant defense, and ECM remodeling. In aged skin, these processes are compromised by cellular senescence, oxidative stress, chronic low-grade inflammation, and structural deterioration of both dermal and epidermal compartments, collectively impairing wound healing. Under these conditions, zinc deficiency may further exacerbate delayed re-epithelialization, defective matrix repair, and persistent inflammation, thereby increasing the risk of chronic wound formation. 

The biological effects of zinc are mediated through multiple interconnected mechanisms, including ZIP and ZnT transporters, MTs, zinc-dependent enzymes, MMPs, and signaling pathways such as GPR39, NF-κB, MAPK, PI3K/AKT, and TGF-β. Taken together, current evidence supports the view that maintaining adequate zinc status may represent an important component of strategies aimed at preserving skin function and improving wound healing in older individuals. However, further experimental and clinical studies are required to define optimal zinc-based interventions, identify responsive patient populations, and clarify the mechanistic links between zinc homeostasis, cellular senescence, and impaired tissue repair.

## Data Availability

No new data were created or analyzed in this study. Data sharing is not applicable to this article.
